# Filamentary Resistive Switching and Capacitance-Voltage Characteristics of the a-IGZO/TiO_2_ Memory

**DOI:** 10.1038/s41598-020-66339-5

**Published:** 2020-06-09

**Authors:** Kwan-Jun Heo, Han-Sang Kim, Jae-Yun Lee, Sung-Jin Kim

**Affiliations:** 1 College of Electrical and Computer Engineering, Chungbuk National University, Cheongju, 28644 Korea; 2 R&D center, SK hynix, 2091, Gyeongchung-daero, Bubal-eup, Icheon-si, Gyeonggi-do, 13558 Korea

**Keywords:** Engineering, Materials science, Nanoscience and technology

## Abstract

In this study, molybdenum tungsten/amorphous InGaZnO (a-IGZO)/TiO_2_/n-type Si-based resistive random access memory (ReRAM) is manufactured. After deposition of the a-IGZO, annealing was performed at 200, 300, 400, and 500 °C for approximately 1 h in order to analyze the effect of temperature change on the ReRAM after post annealing in a furnace. As a result of measuring the current-voltage curve, the a-IGZO/TiO_2_-based ReRAM annealed at 400 °C reached compliance current in a low-resistance state, and showed the most complete hysteresis curve. In the a-IGZO layer annealed at 400 °C, the *O*_1_/*O*_*total*_ value increased most significantly, to approximately 78.2%, and the *O*_3_/*O*_*total*_ value decreased the most, to approximately 2.6%. As a result, the a-IGZO/TiO_2_-based ReRAM annealed at 400 °C reduced conductivity and prevented an increase in leakage current caused by oxygen vacancies with sufficient recovery of the metal-oxygen bond. Scanning electron microscopy analysis revealed that the a-IGZO surface showed hillocks at a high post annealing temperature of 500 °C, which greatly increased the surface roughness and caused the surface area performance to deteriorate. Finally, as a result of measuring the capacitance-voltage curve in the a-IGZO/TiO_2_-based ReRAM in the range of −2 V to 4 V, the accumulation capacitance value of the ReRAM annealed at 400 °C increased most in a nonvolatile behavior.

## Introduction

The unit cell size of the currently developed dynamic random access memory (DRAM) is gradually decreasing for high integration in the manufacturing process^1–3^. This DRAM miniaturization increases difficulty in the capacitor process, and makes it challenging to develop highly integrated cells^4,5^. NAND flash memory, which is nonvolatile memory, faces limitations in terms of control and yield in production because of the complex manufacturing method and the increased number of processes resulting from the structural change from 2D to 3D^6–8^. Consequently, resistive random access memory (ReRAM) is drawing attention as a next-generation memory that overcomes the disadvantages of previous types of memory and has the advantages of high density, nonvolatility, and speed^9–11^. In particular, ReRAM has an access time approximately 10^5^ times faster than flash memory. It can accept a low voltage, similar to DRAM, and can be produced in a simple metal-insulator-metal (MIM) structure^12–15^. The resistance variation characteristic of ReRAM is caused by the dissolution and movement of metal ions based on electrochemical migration at the interface. As a major material for memory motion control, titanium dioxide (TiO_2_), which has high conductivity, is mainly being researched^16–18^. Meanwhile, TiO_2_ implements switching motions using oxygen vacancy, which plays the role of a carrier. However, it has the problem of aggravating the endurance characteristic of memory because of the generation/recombination between oxygen vacancies in the movement process, which causes carrier trapping, reduces the on/off ratio, and lowers electron mobility^14,19–21^.

The electron movement of amorphous indium-gallium-zinc oxide (a-IGZO), which is an oxide semiconductor, is generated through ns-orbitals, in general, and a-IGZO is regarded as a very promising electronic material that can overcome the shortcomings of TiO_2-*x*_. It can obtain fast electron mobility, even in an amorphous state, and can implement excellent resistance switching characteristics in memory^22–24^. However, a-IGZO thin films produced by low-temperature sputtering require post-heat treatment to lower contact resistance at the interface between the electrode and the oxide semiconductor layer^25,26^. The post-heat-treatment process can affect the reduction of the electron trap density and improve electrical performance (such as electron mobility) by removing defects at the semiconductor interface, and increasing the binding force between metals and oxides in the semiconductor^27–29^.

In this study, therefore, memory with a heterojunction structure is manufactured using an a-IGZO oxide semiconductor as an active layer with excellent switching characteristics based on fast electron mobility and a high on/off ratio, compared with TiO_2-*x*_. The electrical, surface, and component effects (based on temperature differences) were examined to determine the most suitable post-heat-treatment condition of the a-IGZO oxide semiconductor. Finally, the capacitance-voltage (C-V) characteristics were observed in order to investigate the characteristics of ReRAM in a metal-insulator-oxide-semiconductor (MIOS) structure based on the manufactured a-IGZO.

## Experimental Section

**Figure 1:**
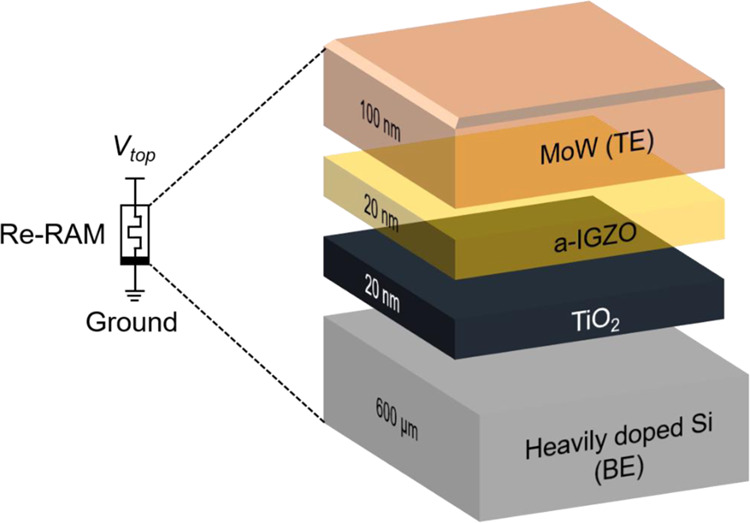
Structure of a-IGZO/TiO_2_-based ReRAM manufactured with post annealing in a furnace at 200, 300, 400, and 500 °C.

Figure 1 shows the structure of the molybdenum tungsten (MoW)/a-IGZO/TiO_2_/n-type silicon (Si)-based MIOS ReRAM manufactured in this study. The electric symbols indicate that the ReRAM is a nonlinear passive device related to the combination of charge and magnetic flux^30,31^. A 600 μm-thick heavily doped n-type Si wafer was used as the substrate and bottom electrode. To remove the surface defects (metallic defects, organic particles), SPM cleaning for 60 m at 90 °C was performed with a solution of sulfuric acid and deionized water at a 3:1 ratio. To produce an insulation membrane on the Si bottom electrode, TiO_2_ was deposited using an atomic layer deposition (ALD) system (LUCIA D100, NCD Co., Ltd., Daejeon, South Korea). For the ALD cycle, titanium tetraisopropoxide was injected as a precursor to Ti. One cycle was defined as dinitrogen (N_2_) purge gas injection for 10 s, water (H_2_O) injection for 1 s, and N_2_ purge gas injection for 10 s. Then, a 20 nm-thick TiO_2_ thin film was formed by repeating 1,000 cycles with 0.2 Å deposition per cycle.

To form an oxide semiconductor, a 20 nm-thick a-IGZO thin film was deposited using a radio frequency (RF) magnetron sputtering process based on a target composed of In:Ga:Zn at a composition ratio of 1:1:1 mol%. After the deposition was complete, heat treatment was applied for approximately 1 h in a furnace to control interface defects and improve crystallinity, thus reducing the interface resistance and increasing the charge concentration and electron mobility^32–34^. To determine the temperature for optimizing the characteristics of a-IGZO-based ReRAM, the furnace temperature was evaluated, and heat treatment was applied for approximately 1 h at temperatures of 200, 300, 400, and 500 °C. After that, a 100 nm-thick MoW source/drain electrode was deposited using DC magnetron sputtering with a shadow mask on top of the a-IGZO. The finally formed upper MoW electrode had a square shape with a length and width of 100 × 100 μm, and four ReRAM devices were put on one wafer. To examine the electrical performance of the finally manufactured a-IGZO/TiO_2_ ReRAM, the current-voltage (I-V) curve was measured. In addition, composition and surface analysis was performed to determine the optimal post-heat-treatment temperature of the a-IGZO oxide semiconductor applicable to ReRAM, and the memory characteristics were checked through C-V measurement.

## Results and Discussion

**Figure 2:**
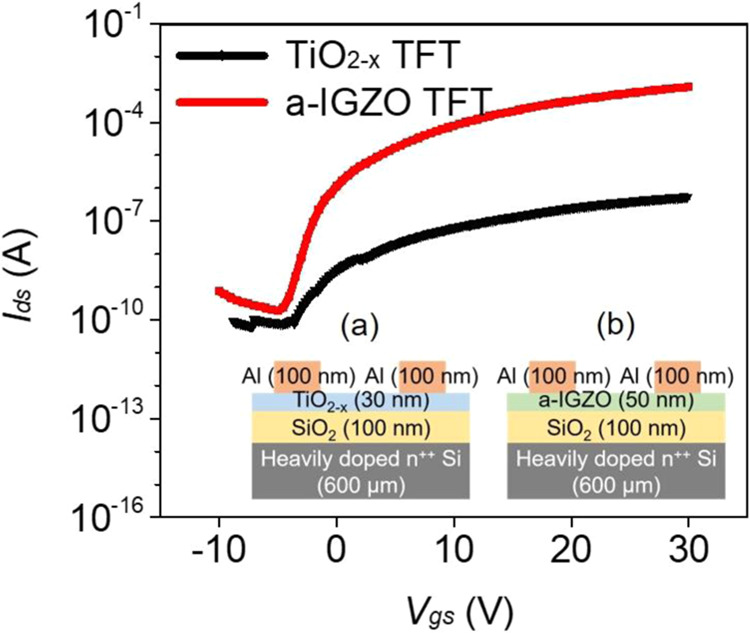
Transfer characteristics of the TiO_2-*x*_ TFT and the a-IGZO TFT with *V*_*ds*_ = 30 V. (inset: structures of the (**a**) TiO_2-*x*_ TFT and (**b**) a-IGZO TFT).

Figure 2 shows the transfer curve graph of the TiO_2-*x*_ thin-film transistor and a-IGZO TFT formed in a top-contact-bottom-gate structure. The insets in Fig. 2a,b show schematics of the TiO_2-*x*_ TFT and a-IGZO TFT from which the transfer curves were extracted. A 600 µm-thick heavily doped n-type Si wafer was used as the substrate and gate bottom electrode. For the TFT, 100 nm-thick SiO_2_ was used as an insulation membrane. The thickness of the TiO_2-*x*_ was 30 nm, and after deposition with ALD, rapid thermal annealing was applied at 700 °C for 5 min. The a-IGZO had a thickness of 50 nm, and after deposition with RF magnetron sputtering, it was heat-treated at 350 °C for 1 h in a furnace. Every electrode was 100 nm-thick aluminum (Al), and the channel length and width were 200 μm and 2,000 μm, respectively.

Bias voltage was applied at 30 V of *V*_*ds*_, and the *I*_*ds*_ value was examined when *V*_*gs*_ was applied from −10 V to 30 V in increments of 0.2 V. According to Table 1, the electron mobility of the a-IGZO TFT was 11.02 cm^2^/Vs, which is excellent, compared with the 0.15 cm^2^/Vs of the TiO_2-*x*_ TFT. Furthermore, the on/off current ratio values showed a large difference at 6.2 × 10^6^ and 7.2 × 10^3^, respectively. The off-current level of the a-IGZO TFT was approximately 1.0 × 10^−10^, which is similar to that of the TiO_2-*x*_ TFT, but the on-current level was approximately 1.0 × 10^−3^, in contrast to the 7.0 × 10^−7^ of the TiO_2-*x*_ TFT. Thus, the a-IGZO TFT is expected to be excellent for implementing high- and low-resistance states (HRS/LRS) characteristic of ReRAM.

**Table 1:** Comparison of the electrical parameters of the TiO_2-*x*_ TFT and the a-IGZO TFT.

Active-layer materials	*μ*_*sat*_ (cm^2^/Vs)	On/off current ratio	*V*_*th*_ (V)	*S*/*S* (V/dec)
TiO_2-*x*_	0.15	7.2 × 10^3^	0.40	0.31
a-IGZO	11.02	6.2 × 10^6^	4.94	0.87

**Figure 3:**
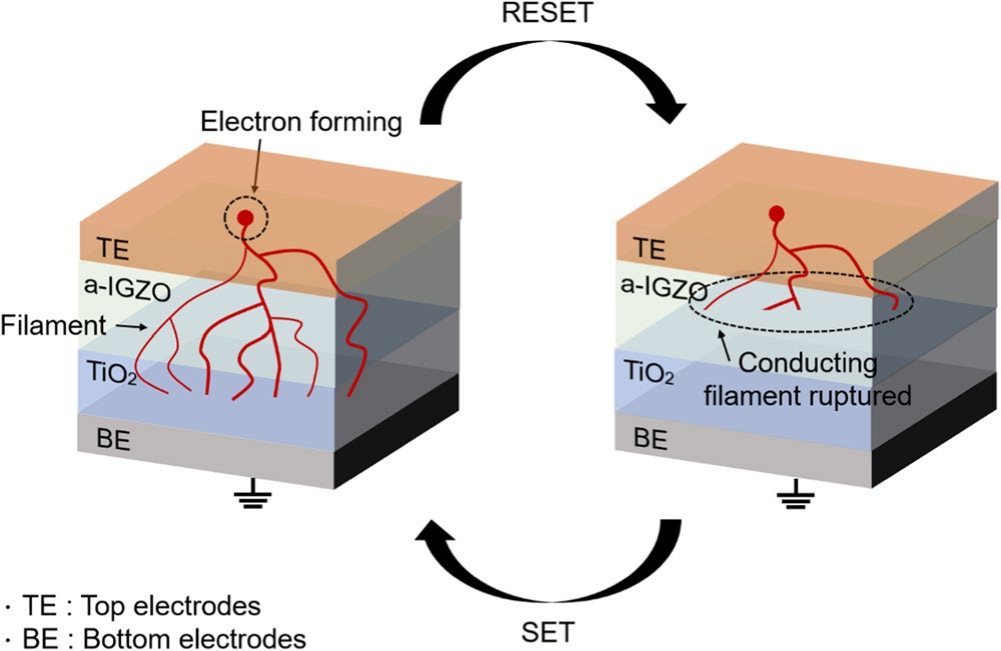
Typical switching mechanism of a-IGZO/TiO_2_-based ReRAM.

Figure 3 illustrates the typical switching mechanism of the a-IGZO/TiO_2_-based ReRAM. For measurement of the ReRAM, a heavily doped n-type Si wafer substrate was used as the bottom electrode, and operation of the a-IGZO ReRAM was checked by applying a voltage to the upper MoW electrode. At first, the current was low when a positive (+) voltage was applied from the a-IGZO oxide active layer to the positive bias area, but the current increased sharply when a specific threshold voltage was applied. This process is called the SET operation^35–37^. By contrast, when a voltage is applied from the positive bias area to the negative bias area, the device reaches the (−) threshold voltage, and the current decreases sharply. This process is called the RESET operation^35,36^. When the state of the a-IGZO thin film reaches the (+) threshold voltage for this process in ReRAM, a conducting pathway is generated because of filament formation^38–40^. However, when a (−) voltage is applied, the oxygen trap of the TiO_2_ insulation layer blocks the electron movement, causing a rupture of the filament^39,40^. Through this process, the resistance state changes according to the applied bias, which induces hysteresis^41–43^ and drives the a-IGZO/TiO_2_ ReRAM.

**Figure 4:**
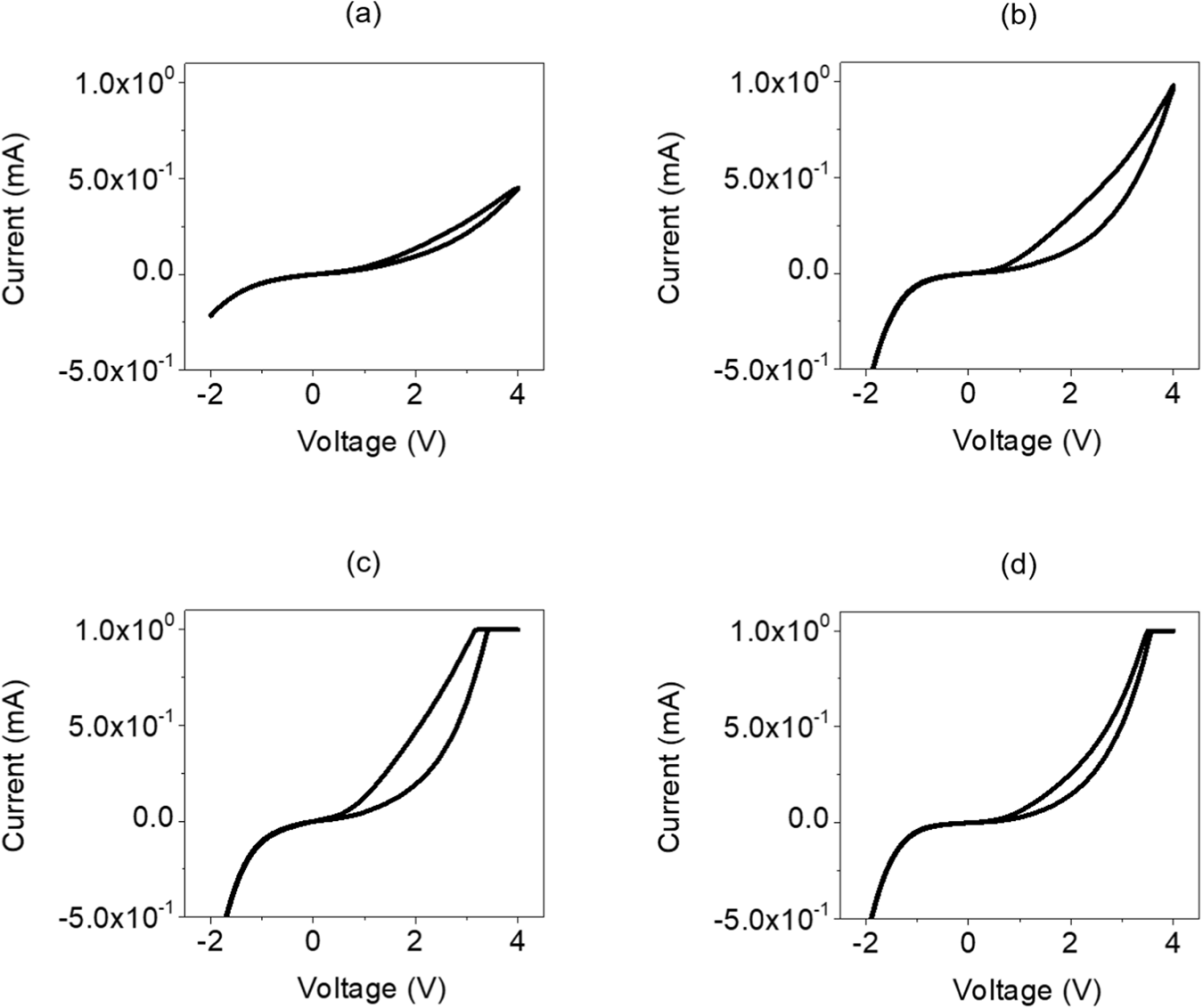
I-V curves for −2 V to 4 V in the a-IGZO layers manufactured with post annealing in a furnace at (**a**) 200 °C, (**b**) 300 °C, (**c**) 400 °C, and (**d**) 500 °C.

Figure 4 shows the measurements of electrical performance from the a-IGZO/TiO_2_-based ReRAM when post-heat treatment was applied at 200, 300, 400, and 500 °C after deposition of the a-IGZO oxide semiconductor layer. For the measurement device, a System 4200 Source Meter (Keithley) was used to measure the I-V curve when −2 V to 4 V was applied. Figure 4a shows the I-V curve of the ReRAM when post-heat treatment was applied at 200 °C. At voltages from 0 V to 4 V, an HRS was observed. Then, at 4 V, which is a reverse sweep, a current of approximately 2.83 × 10^−4^ was recorded, creating an LRS. After reaching the write state, the ReRAM changed again to an HRS after −2 V was applied, and then it changed to the erase state. Figure 4b shows the I-V curve of the ReRAM that underwent post-heat treatment at 300 °C. The current was approximately 9.82 × 10^−4^ at 4 V. In Fig. 4c,d, which are the I-V curves of the ReRAM that underwent post-heat treatment at 400 °C or more, the current increased to 1.00 × 10^−3^, which is set as compliance.

In addition, as the post-heat-treatment temperature of ReRAM was increased, the current level of the LRS increased due to crystallization of the a-IGZO thin film. Through this, it was confirmed that the hysteresis curve characteristics of the memory had been implemented. The ReRAM that underwent post-heat treatment at 200 °C did not show the perfect shape of the hysteresis curve in the (+) area. Using 300 °C created a hysteresis shape with a small width. By contrast, when the post-heat-treatment temperature was 400 °C, the best hysteresis shape was seen. However, the I-V curve of the ReRAM that underwent post-heat treatment at 500 °C reached the compliance current in Fig. 4d, but the hysteresis characteristic decreased as the off-current level increased, and the gap between the HRS and LRS decreased.

In the low range of the post-heat-treatment temperature, electrons were trapped because of interface resistance and the defects on the surface of the a-IGZO thin film. As the temperature increased, the trap density decreased, and as the carrier concentration increased^44–46^, the memory characteristics improved. Therefore, when the post-heat-treatment temperature was 400 °C for the a-IGZO oxide active layer deposited at a low temperature by RF magnetron sputtering, the optimal characteristics of the ReRAM were confirmed through the reduction of the carrier concentration and the trap density of electrons. By contrast, when heat treatment was applied at 500 °C or higher, the hysteresis characteristic decreased because of the unstable state that was formed in some crystals in the IGZO thin film^47–49^.

**Figure 5:**
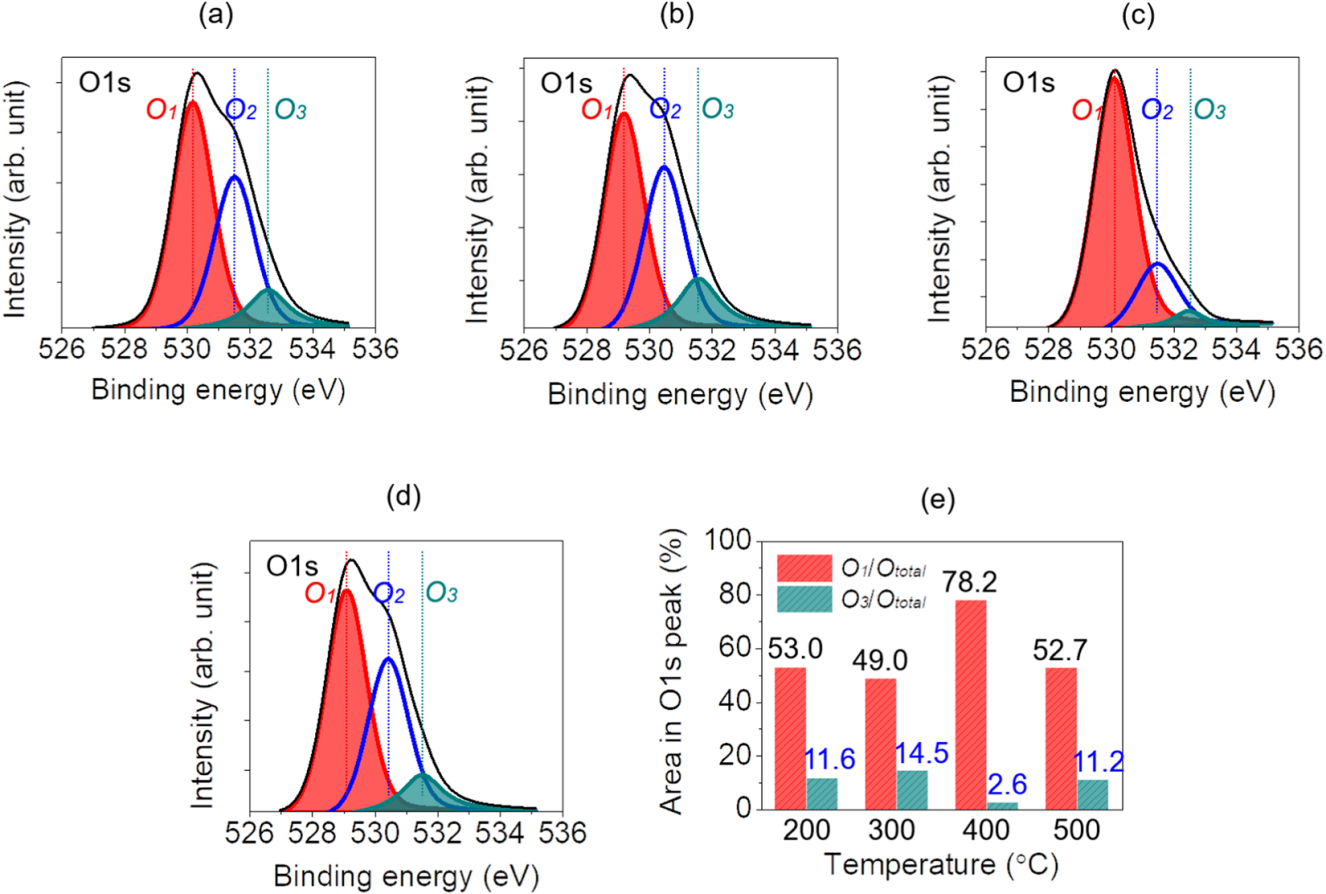
XPS spectra O1s patterns of a-IGZO layers manufactured with post annealing in a furnace at (**a**) 200 °C, (**b**) 300 °C, (**c**) 400 °C, and (**d**) 500 °C, respectively, and (**e**) an area in the O1s peak curve.

Figure 5 shows the x-ray photoelectron spectroscopy (XPS) analysis result of the O1s peaks in the surface after post-heat treatment at 200, 300, 400, and 500 °C after deposition of the a-IGZO oxide semiconductor layer. When post-heat treatment was applied at 400 °C, which is the temperature condition that showed the best electrical performance, the proportion of the oxygen vacancy peaks (*O*_1_) among the O1s peaks was the highest at approximately 78.2%, and the proportion of hydroxide peaks (*O*_3_) was the lowest at approximately 2.6%, as shown in Fig. 5c,e. By contrast, in the composition of the a-IGZO thin film that underwent post-heat treatment at 200 °C, the proportions of *O*_1_ and *O*_3_ among all O1s peaks were approximately 53.0% and 11.6%, respectively, as shown in Fig. 5a,e. Furthermore, in the composition of the a-IGZO thin film that underwent post-heat treatment at 300 °C, the proportion of *O*_1_ among the O1s peaks was approximately 49.0%, as shown in Fig. 5b,e. When compared with the 200 °C condition, the proportion of *O*_1_ decreased slightly from approximately 53.0% to 49.0%, and the proportion of *O*_3_ increased from approximately 11.6% to 14.5%.

As shown above, when the *O*_1_ peak is small and the *O*_3_ peak is large, oxygen vacancies remain without sufficient recovery of the metal-oxygen bonds, and the overall electrical performance of the ReRAM decreases because of the increase in leakage current^50–52^. Furthermore, when the *O*_3_ peak increases, hydrogen ions are combined with oxygen vacancies, and oxygen traps that are relatively strongly bonded with hydrogen increase. As a result, more interface trap charge phenomena that interfere with electron movement occur^53–55^. This is a cause for reduced electron mobility in memory devices while assisting the generation of leakage current, thus lowering the electrical performance of the ReRAM^56–58^.

Meanwhile, when the post-heat-treatment temperature was higher than 500 °C, as shown in Fig. 5d,e, the proportion of *O*_1_ among the O1s peaks in the composition of the a-IGZO thin film decreased significantly (to approximately 52.7%) while the proportion of *O*_3_ increased again to approximately 11.2%. When the proportion of *O*_1_ decreases and the proportion of *O*_3_ increases, the oxygen molecules released from the metal-oxygen bonds quickly diffuse outside the thin film, where the concentration is relatively low, thus causing a decrease in the electrical performance of the ReRAM^50,59,60^. Consequently, this is the same as the electric experiment result that proved the post-heat-treatment temperature of 400 °C is optimal, and it can also be a basis for proving it.

**Figure 6:**
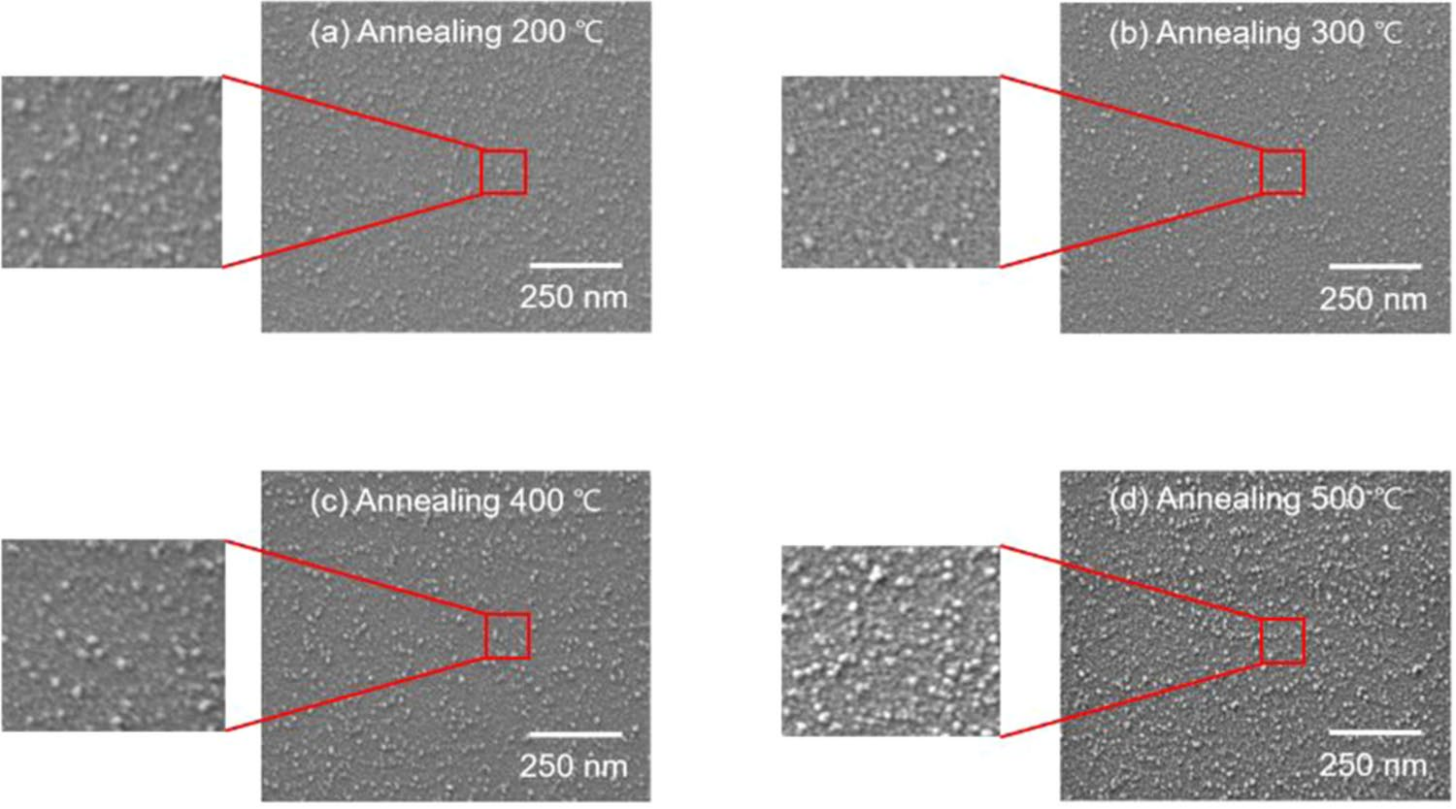
SEM images (50k× magnification) of the a-IGZO layers manufactured by post annealing in a furnace at (**a**) 200 °C, (**b**) 300 °C, (**c**) 400 °C, and (**d**) 500 °C. (inset: enlarged SEM images).

Figure 6 shows measurements using scanning electron microscopy (SEM) with the electron high tension set to 1.0 kV, the I probe set to 1.0 nA, and the working distance set to 2.5 mm in order to analyze the surface area performance of a-IGZO that underwent post-heat treatment at 200, 300, 400, and 500 °C. As shown in Fig. 6a–c, as the post-heat-treatment temperature gradually increased from 200 °C to 400 °C, the grain boundary of the small oval grains grew in size with random curved shapes. However, when post-heat treatment was performed at 500 °C, as shown in Fig. 6d, the large and small grains coexisted on the same surface, and secondary recrystallization^61,62^ was observed^63,64^. This implies that specific grains coarsen more quickly than others in the growing process. This occurs often when there are many impurities or crystals on the surface, and this ultimately causes a decrease in the electrical performance of the ReRAM^7,64,65^.

Furthermore, as the post-heat-treatment temperature increases, the grain boundary becomes more distinct. In the a-IGZO thin film that underwent post-heat treatment at 500 °C, many hillocks were observed on the surface of the thin film. It is presumed that, because of the high post-heat treatment of 500 °C, the degree of relaxation in the compressive stress that the a-IGZO thin film received increased, and small deformations increased, thus increasing the size of the hillocks^66,67^. Therefore, in the a-IGZO thin film that underwent post-heat treatment at 500 °C, the roughness of the surface also increased significantly, because many hillocks were generated. When the surface roughness of the oxide semiconductor is large, a large current leakage occurs because of the interface trap charge phenomenon that interferes with electron movement, resulting in a large, adverse effect on the general electrical properties of the device^68–70^. Consequently, when a high post-heat-treatment temperature like 500 °C is applied after deposition of the a-IGZO oxide semiconductor layer, an aggravated surface state can be seen, which suggests that post-heat treatment at 500 °C or higher has a significant effect on performance degradation of the ReRAM.

**Figure 7:**
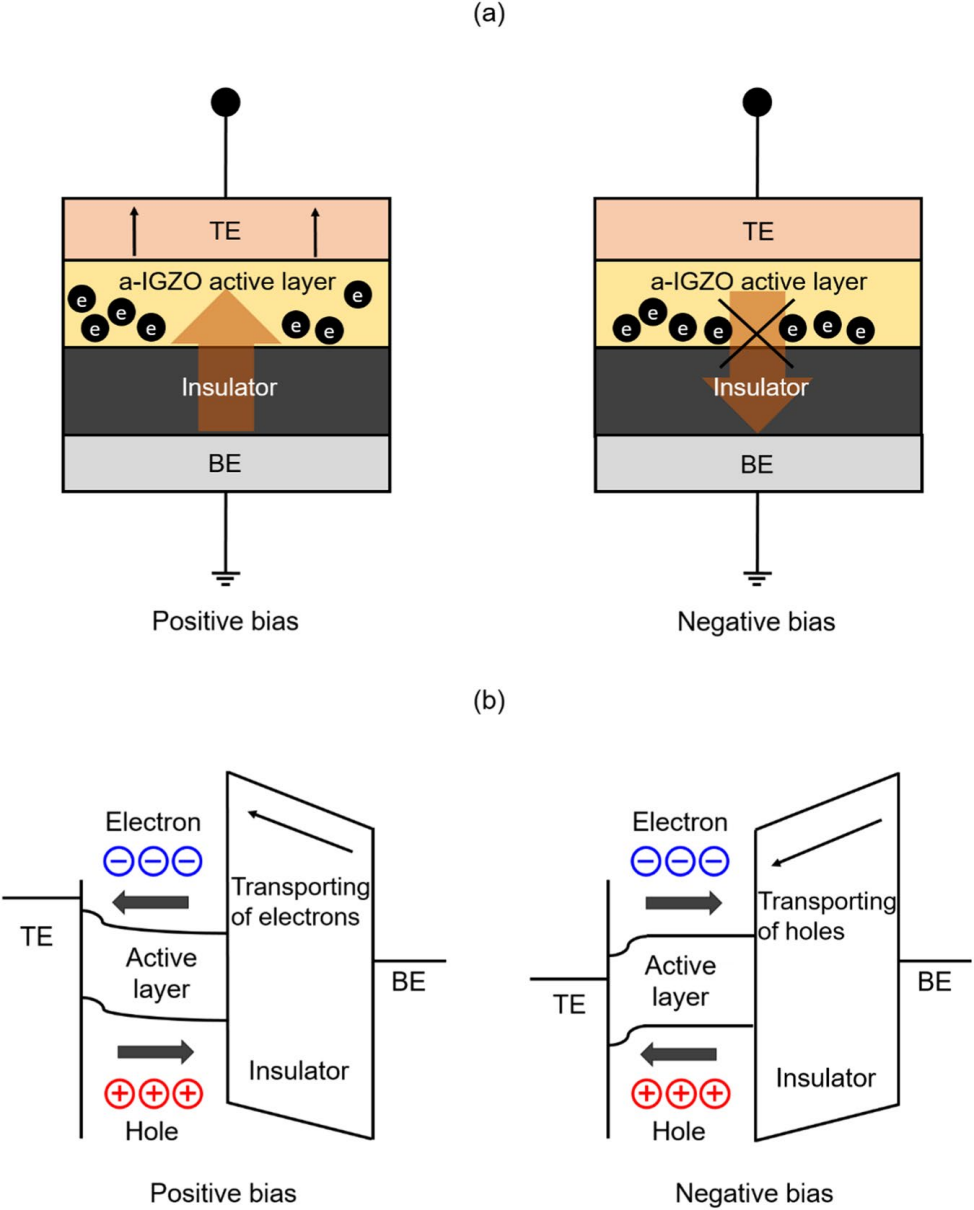
(**a**) Schematics to show that the excited charge carriers in the a-IGZO layer with thermal annealing contribute to the current rise; and (**b**) energy band diagrams representing electron transfer by excited electrons when each positive or negative bias is applied to the upper electrode in the MIOS structure.

Figure 7a shows schematics of the electron movement mechanism in the MIOS structure, analyzing the effect of post-heat treatment of the a-IGZO. The current’s size and direction in the C-V characteristics of the ReRAM that was post-heat treated for a-IGZO depend greatly on the state of the active layer^71^. In a typical MIM device, the electrons injected through the interface of the insulation layer from the top/bottom metal electrodes flow in two directions, and the direction is determined by the applied bias voltage^72–74^. However, the MIOS device flows in a single direction toward the a-IGZO active layer from the metal to which the positive bias voltage is applied^75,76^.

First, the a-IGZO active layer creates an electron movement path by moving the electrons injected by positive voltage applied to the upper MoW metal electrode, and electrons are injected from the interface of the a-IGZO/TiO_2_ through the accumulation area of electrons^77^. The injected electrons form a filament, owing to the conductivity of the bottom electrode, and drive the memory^14,78^. By contrast, when a negative bias voltage is applied to the upper electrode, the electron movement path for the electrons delivered by the TiO_2_ insulating film from the bottom electrode is blocked by the depletion of electrons acting as a capacitor for the TiO_2_-SiO_2_ interface^79,80^. Consequently, the current is halted by the negative bias without flowing, and the filament of the TiO_2_/a-IGZO is ruptured^12,81^. Figure 7b represents the movement of excited electrons as an energy band when a positive or negative bias is applied to the MIOS structure. The electrons and holes changed to an excited state by the voltage applied to the upper electrode, and the excited electrons or holes have the energy for tunneling the TiO_2_ insulating film^82,83^.

**Figure 8:**
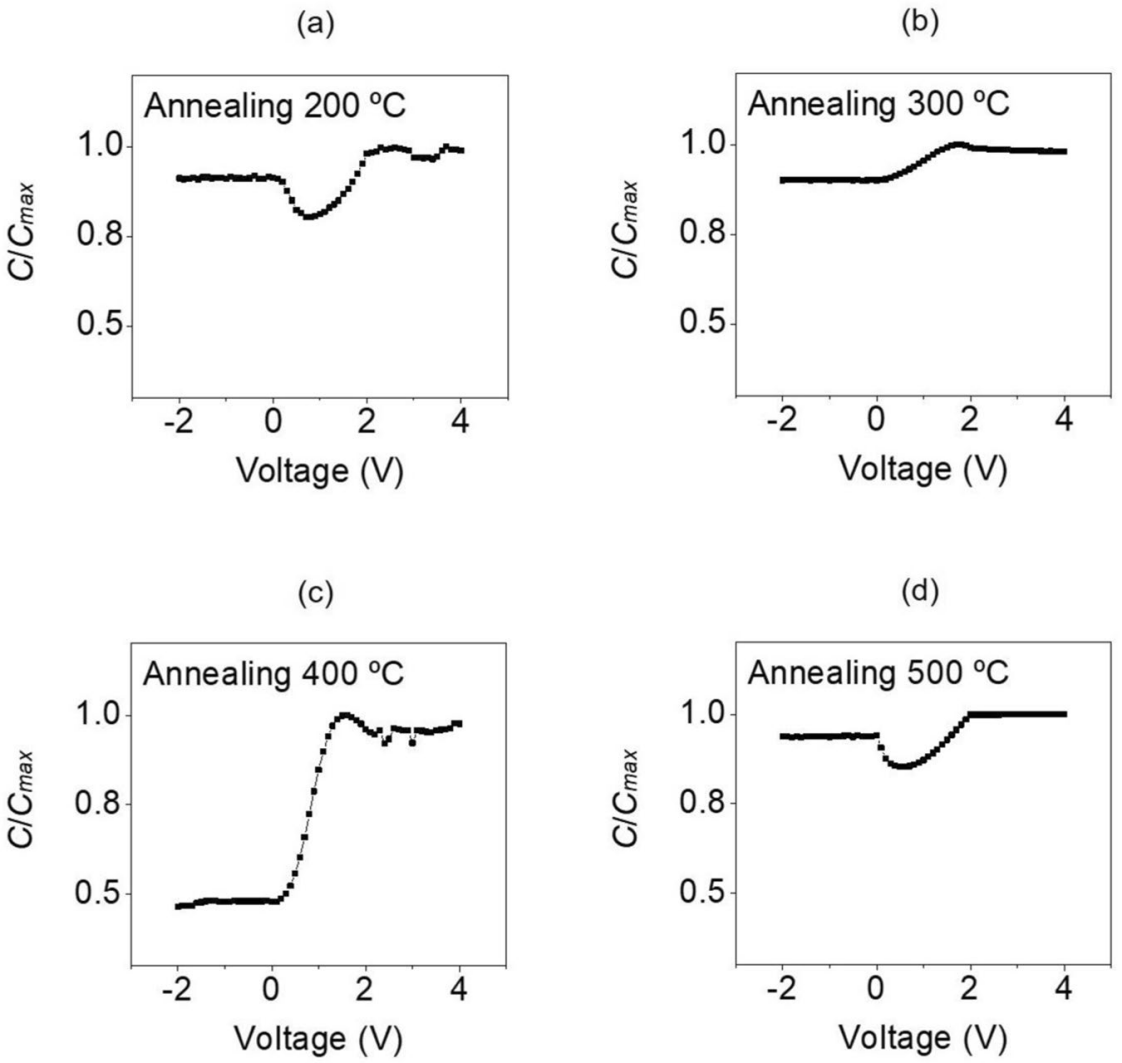
C-V curves for −2 V to 4 V in the a-IGZO layers manufactured with post annealing in a furnace at (**a**) 200 °C, (**b**) 300 °C, (**c**) 400 °C, and (**d**) 500 °C.

Figure 8 shows the measurement results of the C-V curves in the range of –2 V to 4 V in 0.1 V increments for the a-IGZO/TiO_2_-based ReRAM that underwent post-heat treatment at 200, 300, 400, and 500 °C after deposition of the a-IGZO oxide semiconductor layer. The frequency was set to 1 kHz, and the *y*-axis represents *C*/*C*_*max*_, which is the capacitance divided by the maximum capacitance for the applied voltage. As shown in Fig. 8a, the *C*/*C*_*max*_ value was approximately 0.91 in the inversion area for the ReRAM that underwent post-heat treatment at 200 °C, but it was approximately 0.99 in the accumulation area, thus showing a very small difference. Furthermore, in Fig. 8b,d, which show the ReRAM that underwent post-heat treatment at 300 °C and 500 °C, respectively, the *C*/*C*_*max*_ value was approximately 0.90 in the inversion area and 0.98 in the accumulation. Thus, there was little difference from post-heat treatment at 200 °C. Figure 8a,b,d show that the C-V characteristics were not realized due to the inability to optimize the carrier concentration and electron trap density of the a-IGZO thin film caused by a lack of and excess heat treatment. This is because the electrical properties of the memory due to the interfacial trap deteriorate when the recovery of the metal-oxygen bond by heat treatment is not sufficiently achieved. The C-V characteristics of MIOS do not appear to be implemented under 200, 300, and 500 °C because of the inappropriate post-heat treatment temperatures.

However, as shown in Fig. 8c, for the ReRAM that underwent post-heat treatment at 400 °C, the *C*/*C*_*max*_ value was approximately 0.45 when -2 V to 0 V was applied to the upper MoW electrode, indicating that the current state was the inversion section. At this time, the electric field that penetrated through the oxide layer became large enough to induce a small number of carriers to the semiconductor surface. The electron concentration of the semiconductor surface remained relatively constant, because it cannot respond to the high-frequency signals^84,85^. When 0 V to 2 V was applied, because the applied voltage changed from negative (−) to positive (+), the electron energy in the MoW electrode decreased, and (+) charges gathered at the interface between the electrode and oxide. As a result, corresponding (−) charges gathered near the interface between the semiconductor and oxide, which drove out holes or recombined with them, and the interface became a depletion area with no carriers^86,87^. When 2 V to 4 V was applied, the *C*/*C*_*max*_ value became approximately 0.98, and electrons gathered at the interface between the metal and the oxide semiconductor layer, reaching the accumulation area, where the capacitance value increased^88,89^. In conclusion, we verified through the C-V value that the memory characteristics are optimized when a positive or negative bias voltage is applied to the MoW electrode of the a-IGZO-based ReRAM that underwent post-heat treatment at 400 °C.

## Conclusions

MoW/a-IGZO/TiO_2_/n-type Si-based ReRAM was manufactured using ALD and a DC/RF magnetron sputtering system. To analyze the effect of the change in temperature for post-heat treatment using a furnace after depositing a-IGZO (an oxide semiconductor layer) on the a-IGZO/TiO_2_-based ReRAM, the ReRAM was heat-treated for approximately 1 h at 200, 300, 400, and 500 °C. The measurement of the I-V curve showed that the a-IGZO/TiO_2_-based ReRAM that underwent post-heat treatment at 400 °C showed the most perfect hysteresis curve. Furthermore, when the O1s peaks of the a-IGZO thin film were checked through XPS, the a-IGZO thin film that underwent post-heat treatment at 400 °C showed the largest increase in the *O*_1_/*O*_*total*_ value at approximately 78.2%, and the largest decrease in the *O*_3_/*O*_*total*_ value at approximately 2.6%. As a result, the a-IGZO/TiO_2_-based ReRAM that underwent post-heat treatment at 400 °C showed a decrease in conductivity, and prevented the increase in leakage current because the metal-oxygen bonds sufficiently recovered. SEM analysis showed that hillocks are generated on the a-IGZO surface at the high post-heat treatment temperature of 500 °C, thus greatly increasing the surface roughness, and worsening the surface area performance. Finally, measurement of the C-V curve in the range of −2 V to 4 V for the a-IGZO/TiO_2_-based ReRAM confirmed that the memory characteristics were optimized. Therefore, the results of this study prove that when post-heat treatment is applied to the a-IGZO oxide active layer, 400 °C produces the best electrical performance in the ReRAM, and the best composition and surface state in the a-IGZO thin film.
